# Selbsthilfe und Gesundheitskompetenz während der Corona-Pandemie: Ergebnisse der qualitativen GeMSe^HeCo^-Studie

**DOI:** 10.1007/s11553-021-00881-6

**Published:** 2021-07-26

**Authors:** Lisa Marie Kogel, Natalie Steeb, Katharina Rathmann

**Affiliations:** Fachbereich Pflege und Gesundheit, Public Health Zentrum Fulda (PHZF), Hochschule Fulda, Fulda, Deutschland

**Keywords:** Gesundheitsinformationen, Menschen mit chronischer Erkrankung, Behinderung, Selbsthilfegruppen, Covid-19, Health Information, Chronic disease, Disability, Self-help groups, Covid-19

## Abstract

**Hintergrund:**

Die Pandemie stellt Menschen mit Beeinträchtigung vor vielfältige Herausforderungen, insbesondere auch beim Umgang mit Gesundheitsinformationen. Bislang ist nur wenig über die Rolle der Selbsthilfe bei der Förderung der Gesundheitskompetenz ihrer Mitglieder während der Pandemie bekannt.

**Methodik:**

Der Studie „Gesundheitskompetenz von Mitgliedern der Selbsthilfe: Herausforderungen durch die Corona-Pandemie (GeMSe^HeCo^)“ liegt ein qualitatives Studiendesign zugrunde. Der Feldzugang erfolgte deutschlandweit über die Selbsthilfegruppen. Von Juli bis Oktober 2020 wurden *N* = 19 Telefoninterviews mit Vertreter*innen von Selbsthilfegruppen durchgeführt, transkribiert und inhaltsanalytisch ausgewertet.

**Ergebnisse:**

Vertreter*innen der Selbsthilfe berichteten über Stärken und Schwächen der Mitglieder im Umgang mit Gesundheitsinformationen während der Corona-Pandemie. Schwächen wurden v. a. bei älteren Mitgliedern, Menschen mit niedrigem Bildungsniveau und mit Migrationshintergrund berichtet. Stärken beobachteten die Vertreter*innen bei langjährigen Mitgliedern und bei Mitgliedern, die sich zu ihrer Krankheit kontinuierlich weiterbilden.

**Diskussion:**

Die Selbsthilfe wird während der Pandemie als wichtige Unterstützung der Mitglieder im Umgang mit Gesundheitsinformationen wahrgenommen. Ein Vorteil für die Mitglieder besteht insbesondere in der Prüfung und Zusammenstellung von Gesundheitsinformationen nach individuellen Bedarfen durch die Selbsthilfe. Herausforderungen können aufgrund von Sprach- und Verständnisschwierigkeiten entstehen, bspw. durch einen Migrationshintergrund. Der Ausbau von digitalen Angeboten hat sich in dieser Zeit als hilfreich erwiesen. Dabei sollte die Unterstützung vermehrt Ältere und Mitglieder mit geringen finanziellen Möglichkeiten berücksichtigen, da diese während der Pandemie weniger Zugang zu digitalen Angeboten aufweisen.

## Abstract (en)

**Background:**

The Corona pandemic poses multiple challenges for people with impairments, including managing health information. To date, little is known about the role of self-help in promoting health literacy among its members during the Corona pandemic.

**Methods:**

“The Health Literacy of Self-Help group members: Challenges of the Corona-pandemic (GeMSe^HeCo^)” study is based on a qualitative study design. Field access was provided by self-help advocacy groups throughout Germany. For this purpose, telephone interviews were conducted from July–October 2020 with *N* = 19 representatives of self-help groups, transcribed and content-analyzed.

**Results:**

Self-help group representatives reported strengths and weaknesses in members’ use of health information during the Corona pandemic. Weaknesses were reported primarily among older members, those with low levels of education, and those with immigrant backgrounds. Strengths were observed by the representatives among long-term members and among members who continuously educate themselves about their disease.

**Discussion:**

Self-help groups are perceived as providing important support to members in dealing with health information during the Corona pandemic. One particular benefit to members is the self-help group’s review and compilation of health information according to individual needs. Challenges can arise due to difficulties in German language and understanding, e.g., as a result of a migration background. The expansion of digital offers has proven helpful during this time. In this context, support should increasingly consider older members and members with low financial means, as they have less access to digital offers especially during the Corona pandemic.

Schutzmaßnahmen und Kontaktverbote während der andauernden Corona-Pandemie erschweren persönliche Treffen – insbesondere für Personengruppen mit chronischen Erkrankungen und Beeinträchtigungen, die sich in Selbsthilfegruppen organisieren. Dabei leistet die Selbsthilfe gerade in Krisenzeiten einen wesentlichen Beitrag zur Bewältigung von Herausforderungen und zur Förderung der Gesundheitskompetenz.

Während der Corona-Pandemie sind Selbsthilfegruppen eine zentrale Quelle für die Unterstützung der Mitglieder bei der Bewältigung von gesundheitsbezogenen Problemen. Durch ihr Engagement stellen sie ihren Mitgliedern wichtige Impulse für die Alltagsbewältigung bereit [13]. Der frühere Leitbegriff der gemeinschaftlichen Selbsthilfe, das „Empowerment“, wird jüngst durch den Begriff Gesundheitskompetenz ergänzt [5]. Unter Gesundheitskompetenz werden Fähigkeiten und Fertigkeiten im Umgang mit gesundheitsrelevanten Informationen verstanden, die benötigt werden, um die Gesundheit zu fördern, Krankheit zu bewältigen und das Gesundheitssystem adäquat nutzen zu können [14].

Insbesondere für Menschen mit Beeinträchtigung ist ein kompetenter Umgang mit Gesundheitsinformationen entscheidend, da sie sich täglich mit Informationen bezüglich ihrer Therapie oder Medikamente auseinander setzten müssen [11]. Gesundheitskompetenz ist demnach eine zentrale Fähigkeit, damit das individuelle Krankheitsmanagement und die Vermeidung von Folge- und Späterkrankungen gelingen kann, aber auch, um an Entscheidungsprozessen über medizinische Leistungen teilhaben zu können [3]. Während der Corona-Pandemie, die ohnehin viele Menschen verunsichert, spielen Gesundheitsinformationen und verlässliche Quellen eine besonders wichtige Rolle.

Für die Mitglieder der Selbsthilfe ist es gerade während der Corona-Pandemie von Bedeutung, dass sie im Umgang mit Gesundheitsinformationen unterstützt werden. Hierdurch leistet die Selbsthilfe einen wichtigen Beitrag zur Förderung der Gesundheitskompetenz ihrer Mitglieder. Insbesondere die gesundheitsbezogene Selbsthilfe fördert das Krankheitsmanagement der Betroffenen, bietet Unterstützung im alltäglichen Leben mit der Erkrankung und im Umgang mit Krisensituationen [3]. Selbsthilfeorganisationen sind daher im Sinne der Salutogenese für die Stärkung und Förderung der Gesundheitskompetenz in Deutschland von großer Bedeutung. Durch sie soll ein höheres Maß an Selbstbestimmung und Selbstständigkeit der Menschen mit Beeinträchtigung ermöglicht und ihre Handlungskompetenz gestärkt werden [6]. Durch die eigene Betroffenheit und ein umfangsreiches Erfahrungswissen unterstützen Selbsthilfegruppen auf persönlichere Weise im Vergleich zu Akteuren des Gesundheitswesens [13]. Während der Corona-Pandemie wird eine besondere Herausforderung in der „Infodemie“ und dem eigenverantwortlichen Handeln gesehen, um sich über die Corona-Pandemie zu informieren und adäquat davor schützen zu können [12]. Dazu müssen Informationen über das Virus, die Schutz- und Hygienemaßnahmen und zu den Kontaktregelungen gefunden, verstanden, kritisch bewertet und angewendet werden. Somit wird die Gesundheitskompetenz zu einer Kernkompetenz im Umgang mit der Pandemie und ihrer Bewältigung [9]. Welche Herausforderungen sich durch Ausfall der Gruppentreffen für Mitglieder ergeben und welche Rolle die Selbsthilfe zur Unterstützung und Förderung der Mitglieder insbesondere im Umgang mit Gesundheitsinformationen während der Corona-Pandemie einnimmt, ist bislang noch nicht untersucht worden.

## Ziele und Fragestellungen

Ziele dieses Beitrags sind:

1. die Gesundheitskompetenz der Mitglieder der Selbsthilfe während der Corona-Pandemie aus Sicht der Vertreter*innen der Selbsthilfe darzustellen,
2. die Rolle der Selbsthilfe bei der Förderung der Gesundheitskompetenz der Mitglieder zu erheben.

Dabei wird folgenden Fragestellungen nachgegangen:

1. Welche Stärken und Schwächen weisen Mitglieder in den verschiedenen Bereichen der Gesundheitskompetenz während der Corona-Pandemie auf?
2. Welche Möglichkeiten zur Förderung der Gesundheitskompetenz durch die Selbsthilfe bestehen in Deutschland?

## Methodik

Um Stärken und Schwächen der Gesundheitskompetenz bei Mitgliedern der Selbsthilfe in Deutschland zu erfassen und die Rolle der Selbsthilfe während der Pandemie zu erheben, wurde ein qualitatives Design in Form von deutschlandweiten Telefoninterviews mit Vertreter*innen der Selbsthilfegruppen angewendet. Die Interviews fanden im Zeitraum von Juli bis Oktober 2020 statt. Die Stichprobengröße umfasste 19 Vertreter*innen der Selbsthilfe, von denen 63,2 % weiblich waren. Das Durchschnittsalter betrug 55,6 Jahre. 73,7 % der Befragte gaben an, selber eine Beeinträchtigung zu haben. Die Interviewdauer betrug durchschnittlich 55 (Min. 28, Max. 127) min. Alle Personen stimmten der Teilnahme an der Studie sowie der Aufzeichnung der Gespräche zu. Die Richtlinien zum Datenschutz wurden in der gesamten Studie eingehalten. Die Beschreibung der Stichprobe kann der Tab. 1 entnommen werden.

**Tab. 1:** Stichprobe (Vertreter*innen der Selbsthilfe in *n* und %)

*Soziodemografische Angaben*		*n (%)*
Geschlecht	Männlich	7 (36,8)
	Weiblich	12 (63,2)
Alter in Jahren (Min.–Max.)		55,6 (31–81)
Beeinträchtigung	Ja	14 (73,7)
	Nein	4 (21,0)
	Keine Angabe	1 (5,3)
*Angaben zur Selbsthilfe*		*n (%)*
Seit wann Vertreter*in der Selbsthilfe in Jahren (Min.–Max.)		9,3 (1–33)
Bundesland (Mehrfachnennungen möglich)	Bremen	1 (4,8)
	Hamburg	2 (9,6)
	Hessen	6 (28,6)
	Niedersachsen	2 (9,6)
	Nordrhein-Westfalen	7 (33,3)
	Rheinland-Pfalz	1 (4,8)
	Sachsen	2 (9,6)
Themengebiet der Selbsthilfe (Mehrfachnennungen möglich)	Behinderung und Elternschaft	1 (4,5)
	Körperliche Erkrankungen	9 (40,9)
	Krebserkrankungen	3 (13,6)
	Lebensbewältigung	3 (13,6)
	Psychische Erkrankungen	4 (18,2)
	Sucht	2 (9,1)
Interviewdauer in Minuten: Durchschnitt (Min.–Max.)		55 (28–127)

*Min–Max* = Spannweite; durch die Möglichkeit der Mehrfachnennungen werden beim Bundesland (*n* = 21) und Themengebiet der Selbsthilfe (*n* = 22) alle Angaben dargestellt. Es ergibt sich daher eine Prozentangabe > 100 %

### Erhebungsinstrument

Der Interviewleitfaden widmet sich den Herausforderungen, die sich für Menschen mit Beeinträchtigung während der Corona-Pandemie ergeben. Im Rahmen der Gemse^HeCo^-Studie wurden die Rolle der Selbsthilfe und das Informationssuchverhalten der Mitglieder bezüglich der Gesundheitsinformationen während der Pandemie erfragt. Hierbei ging es um die Einschätzung der Gesundheitskompetenz der Mitglieder und um bestehende Angebote sowie Unterstützungsmöglichkeiten zur Stärkung der Gesundheitskompetenz während der Pandemie.

### Auswertungsmethodik

Die Transkription der Interviews erfolgte mittels des Programms *F4 Transkript*. Die Auswertung wurde anhand einer qualitativen Inhaltsanalyse nach Kuckartz mit dem Programm MAXQDA der Firma VERBI (Berlin, Deutschland) durchgeführt [7]. Die Datenauswertung folgte einer zirkulären Struktur. Das Material wurde wiederholt gesichtet, zu Haupt- und in einem späteren Schritt zu Subkategorien zugeordnet, paraphrasiert und schließlich verknüpft [7]. Die Kategorien für die Studie wurden sowohl induktiv aus den Erkenntnissen des Interviewmaterials als auch deduktiv aus dem Forschungsstand entwickelt [8]. Durch diese Auswertungsmethode konnte der wesentliche Informationsgehalt aus den Interviews bezogen auf die Forschungsfragen extrahiert werden.

## Ergebnisse

Insgesamt nannten die Vertreter*innen der Selbsthilfe während der Pandemie sowohl Stärken als auch Schwächen im Umgang mit Gesundheitsinformationen bei ihren Mitgliedern. Schwächen im Umgang mit Gesundheitsinformationen wurden v. a. bei älteren Mitgliedern, Menschen mit niedrigem Bildungsniveau und bei Personen mit einem Migrationshintergrund berichtet.

Stärken wurden hingegen bei langjährigen Mitgliedern beobachtet und bei Mitgliedern, die sich zu ihrer Krankheit informieren und kontinuierlich weiterbilden. Tab. 2 stellt exemplarisch Zitate zu den Stärken und Schwächen der Mitglieder im Umgang mit Gesundheitsinformationen dar.

**Tab. 2:** Exemplarische Zitate zum Umgang mit Gesundheitsinformationen der Mitglieder aus Sicht der Vertreter*innen der Selbsthilfe vor und während der Corona-Pandemie

Themenbereich	Exemplarische Zitate
Stärken im Bereich „Finden“	„Ich denke also viele dieser Menschen, die sich mit der Krankheit auseinandersetzten und die sich mit ihrem Krankheitsbild vertraut gemacht haben, haben ihren eigenen Wege gefunden […] wie sie an Gesundheitsinformationen kommen können“ (HP, S. 6: 46–49)
Schwächen im Bereich „Finden“	„Wenn man sucht, [kommt] eine Informationsflut […], dass es einfach zu viel ist und man am Ende dann gar nicht weiß, wo soll ich mich […] hinwenden“ (MM, S. 8: 44–47). „Ich würde sagen […] Überflutung, […] es ist zu viel, es ist undurchsichtig und unüberschaubar und ich glaube, wenn man sowieso schon […] psychisch belastet ist, […] dann ist es einfach sehr mühsam sich durch diesen Informationsdschungel zu kämpfen“ (GD, S. 7–8: 29–3)
Stärken im Bereich „Verstehen“	„Aber sonst das Gesundheitswesen zu verstehen ist glaube ich nicht so der große Punkt“ (BR., S. 11 Z. 40–41). „[Informationen sind an] den Bedarf der Mitglieder angepasst und [werden] auch nur punktgenau weitergegeben, der sie benötigt“ (CD, S. 7: 23–26). „Es gibt diejenigen, die sich eigeninitiativ beschäftigen und auch sprachlich und intellektuell ausreichend geschult sind, dass sie das Verstehen können, auch eine Studie lesen können, dass womöglich auch in Englisch. Dann gibt es die, die sich damit befassen und auch Hintergrundinformationen haben […]. Und dann gibt es die Leute, die gerne mal solche Sachen auch abrufen und nachfragen und hören.“ (SB, S. 12: 9–20)
Schwächen im Bereich „Verstehen“	„Also das sind einmal Sprachhindernisse, dass so viele Informationen in Englisch publiziert werden.“ (SB, S. 9: 8–9). „Ich war vor kurzem […] zu einem Migrantenfrühstück eingeladen […]. Die sind genau so krank […] wie wir Deutschen […]. Nur die Unterstützung da ist bedeutend schwerer, weil […] die Sprache teilweise nicht da ist“ (BR, S. 12: 1–5)
Stärken im Bereich „Beurteilen“	„Weil die Leute, die bei uns dann schon Mitglied sind, [sind] ‚Experten in eigener Sache‘“ (LS, S. 11: 45–46). „Weil auch immer mehr ältere Leute […] digital durchaus sehr gut in der Lage sind […] sich selbst zu allen Zeiten […] damit zu beschäftigen und Informationen zu bekommen. Sie müssen nur eine Unterstützung haben, dass man gemeinsam solche Dinge durchspricht“ (LO, S. 6: 24–29)
Schwächen im Bereich „Beurteilen“	„Da haben heute Morgen schon wieder zwei Leute angerufen, ob sie jetzt auch gefährdet wären. Ja, viele Falschinformationen“ (HE., S. 10, Z. 14–15). „Die klicken fünf Minuten sich durch das Netz, lesen irgendwas und nehmen das für Wahres, das ist das Problem“ (EM, S. 13: 10–11)
Stärken im Bereich „Anwenden“	„Also, das Anwenden [ist] glaub ich nicht [ein Problem] weil das ist eine Gewohnheitssache.“ (BR, S. 14: 46–47). „Bei den Stammmitglieder bin ich mir hundertprozentig sicher, die sind bestens gebrieft“ (CD, S. 12: 25–26)
Schwächen im Bereich „Anwenden“	„[Mitglieder] haben schon Schwierigkeiten den richtigen Arzt oder das richtige Krankenhaus zu finden oder die richtige Reha zu organisieren. Ja, auch große Schwierigkeiten mit den formellen Dingen, wie auch Beantragung von Schwerbehinderung, Beantragung von Reha, Beantragung von Grundsicherung und so weiter“ (SB, S. 11:16–20). „Das [sind] weitgehend […] ganz alte Leute. [...] Wo man eigentlich sagen kann, gut wenn sie das einhalten, was der Arzt ihnen sagt. Aber die jetzt in dem Sinne fortzubilden oder weiter zu informieren, das wird wohl eher nicht gelingen“ (BR, S. 13: 47–50)
Möglichkeiten der Förderung der Selbsthilfe während Corona-Pandemie	„Wir haben […] Telefonaktionen gemacht. Wo wir […] unsere Mitglieder […] angerufen haben […] und dann praktisch den Kontakt gehalten haben“ (BR, S. 4: 18–22). „Wir haben eine Physiotherapeutin, die […] in der Corona-Pandemie jeden Tag ein 15 bis 20 min Video mit Sporteinheiten online gestellt hat“ (WA, S. 10, Z. 37–40)

### Umgang mit Gesundheitsinformation im Bereich „Finden“

Im Bereich „Finden“ von Gesundheitsinformationen berichteten die Vertreter*innen, ihre Mitglieder wüssten, dass sie die benötigten Gesundheitsinformationen zur individuellen Erkrankung aber auch zu der Corona-Pandemie durch die Selbsthilfe erhalten können. Neue Mitglieder hingegen stehen bei der Suche nach Gesundheitsinformationen besonders seit der Pandemie vor Herausforderungen, da ihnen nicht bekannt sei, welche Unterstützung die Selbsthilfe dabei leisten kann und an wen sie sich im Bedarfsfall wenden können. Durch den Ausfall der Selbsthilfetreffen fehlt der persönliche Austausch zu den Vertretungen und betroffenen Mitgliedern. Dabei trägt eine gute Vernetzung der Mitglieder untereinander zur Stärkung der Gesundheitskompetenz bei, da sich Mitglieder gegenseitig bei der Suche nach entsprechenden Gesundheitsinformationen unterstützen können.

Um die Mitglieder während der Pandemie zu unterstützen, werden Gesundheitsinformationen häufig digital und per E‑Mail weitergegeben. Eine Herausforderung entsteht hierbei durch unterschiedlich gute Computerkenntnisse und eine heterogene technische Ausstattung (z. B. Computer oder E‑Mail-Adresse) der Mitglieder.

### Umgang mit Gesundheitsinformation im Bereich „Verstehen“

Im Bereich „Verstehen“ von gesundheitsbezogenen Informationen wurde von den Vertreter*innen der Selbsthilfe berichtet, dass pandemiebezogene Gesundheitsinformationen vor der Weitergabe an die Mitglieder von der Selbsthilfe auf ihre Qualität geprüft und an den Bedarf dieser angepasst werden.

Schon vor der Pandemie bestanden Herausforderungen bei der Informationsweitergabe und Beratung durch Sprachbarrieren bei Mitgliedern mit Migrationshintergrund. Zudem sind Verständnisschwierigkeiten von Fachsprache, welche von ärztlichem Personal, in wissenschaftlichen Artikeln oder Fachliteratur zu gesundheitsrelevanten Themen verwendet wird auch bei deutschsprachigen Mitgliedern bekannt. Auch in der aktuellen Situation werden solche Schwierigkeiten beim „Verstehen“ von Informationen zum Umgang mit der Corona-Pandemie beobachtet.

### Umgang mit Gesundheitsinformation im Bereich „Beurteilen“

Im Bereich „Beurteilen“ von Gesundheitsinformationen berichteten die Vertreter*innen der Selbsthilfegruppen, dass gerade langjährige Mitglieder Therapieerfahrung und Reflexionsvermögen aufweisen und aufgrund des häufigen Umgangs mit neuen Gesundheitsinformationen „Experten in eigener Sache“ sind. Die große Flut an gesundheitsrelevanten Informationen während der Pandemie stellt die Mitglieder dennoch häufig vor neue, große Herausforderungen: Hier wurde berichtetet, dass bei der Beurteilung der Informationen für die eigene Gesundheit oft Verunsicherungen bestehen und eine ausführliche Recherche nach entsprechenden Informationen als sehr zeitaufwändig empfunden wird.

### Umgang mit Gesundheitsinformation im Bereich „Anwenden“

Der Bereich „Anwenden“ der Gesundheitsinformationen stellt für die langjährigen Mitglieder laut vieler Vertreter*innen eine Gewohnheit dar. Durch „Anwenden“ von Informationen zur Verbesserung eigener Krankheitssymptome werden Kompetenzen im Umgang mit Gesundheitsinformationen ausgebildet. Allerdings wird auch berichtet, dass die Mitglieder während der Corona-Pandemie nicht gut durch ihre Selbsthilfegruppe unterstützt werden konnten. Einen wichtigen Aspekt stellt dabei die Einweisung in neue krankheitsspezifische Techniken sowie medizinische Produkte und Hilfsmittel dar. Durch nicht stattgefundene Gruppentreffen und den fehlenden persönlichen Kontakt war es für die Selbsthilfe häufig nicht möglich, die Mitglieder praktisch anzuleiten.

### Möglichkeiten der Selbsthilfe zur Förderung der Gesundheitskompetenz der Mitglieder während der Corona-Pandemie

Um ihre Mitglieder auch während der Corona-Pandemie unterstützen zu können, musste sich die Selbsthilfe intern neu organisieren. Durch Ausfall der Gruppentreffen mussten andere Wege gefunden werden, um den Mitgliedern die Gesundheitsinformationen zugänglich zu machen. Insbesondere webbasierte Angebote, wie Informationen auf der Webseite, im Newsletter, über WhatsApp oder in Social-Media-Kanälen wurden in dieser Zeit durch die Selbsthilfe deutlich ausgebaut und fanden gerade bei jüngeren Mitgliedern Zuspruch. Allerdings verwiesen die Vertreter*innen auch darauf, dass digitale Angebote nicht alle Mitglieder erreichen. Daher wurden ältere Mitglieder und jene mit geringen finanziellen Möglichkeiten durch telefonische Kontaktaufnahme und -angebote unterstützt. Die Vertreter*innen gaben an, dass digitale Alternativen, wie bspw. die Anleitung und Beratung über Video oder Telefon, den persönlichen Kontakt in Präsenz nicht gänzlich ersetzen können.

Gesundheitsinformationen zur Corona-Pandemie wurden vor der Weitergabe an die Mitglieder von der Selbsthilfe auf ihre Relevanz und Qualität geprüft und zum besseren Verständnis an den individuellen Bedarf der Mitglieder angepasst. Dies erforderte v. a. zu Beginn der Pandemie lange, aufwändige Recherchearbeit für die Vertreter*innen von Selbsthilfegruppen.

## Diskussion

Ziele des Beitrags waren es, die Gesundheitskompetenz der Mitglieder der Selbsthilfe während der Corona-Pandemie aus Sicht der Vertreter*innen darzustellen und die Rolle der Selbsthilfe bei der Förderung der Gesundheitskompetenz der Mitglieder zu erfassen.

Bezüglich des ersten Ziels kann festgehalten werden, dass das „Finden“, „Verstehen“, „Beurteilen“ und „Anwenden“ von verlässlichen Gesundheitsinformationen gerade während der Pandemie einen hohen gesundheitlichen Stellenwert für Menschen mit Beeinträchtigung hat. Berichtete Schwierigkeiten der Mitglieder bestehen v. a. in den Bereichen „Finden“ und „Beurteilen“ der gesundheitsbezogenen Informationen. Sie entstehen insbesondere durch die Überflutung mit Informationen zur Corona-Pandemie und der daraus resultierenden Verunsicherung der Mitglieder. Die vorliegenden Ergebnisse verdeutlichen, dass Vertreter*innen häufiger Schwierigkeiten im Umgang mit Gesundheitsinformationen bei Mitgliedern mit niedrigem Bildungshintergrund und Mitgliedern mit Migrationshintergrund feststellen. Diese Zusammenhänge werden auch von Schäfer et al. (2021) beschrieben [12]. Verständnisschwierigkeiten wurden zudem auch bei deutschsprachigen Mitgliedern berichtet, wenn Informationen nur in medizinischer Fachsprache zur Verfügung stehen oder medizinisches Personal Fachsprache benutzt. Vor allem während der Pandemie zeigt sich ein großer Bedarf, Gesundheitsinformationen bezüglich der eigenen Krankheit, aber auch zum Umgang mit dem Coronavirus in anderen Sprachen und leicht verständlich für die Mitglieder zugänglich zu machen. Diese Notwendigkeit zeigt sich auch in anderer Literatur zu Menschen mit Beeinträchtigungen [10].

Die Rolle der Selbsthilfe bei der Förderung ihrer Mitglieder während der Corona-Pandemie wird insbesondere bei der Weitergabe und Vermittlung von gesundheitsrelevanten Informationen aber auch in der Beratung der Mitglieder zu gesundheitlichen Themen deutlich. Die Prüfung und individuelle Zusammenstellung von Gesundheitsinformationen durch die Selbsthilfe werden als förderlich für die Stärkung der individuellen Gesundheitskompetenz der Mitglieder betrachtet [2]. Laut Vertreter*innen führt die Unterstützung der Selbsthilfe dazu, dass langjährige Mitglieder in allen Bereichen der Gesundheitskompetenz besser aufgestellt sind als neue Mitglieder.

## Zusammenfassung

Die vorliegenden Ergebnisse der GeMSe^HeCo^-Studie verdeutlichen, dass die Selbsthilfe während der Pandemie für ihre Mitglieder einen unverzichtbaren Partner im Umgang mit Gesundheitsinformationen darstellt. Die Rolle der Selbsthilfe während der Corona-Pandemie wurde in der Literatur bisweilen wenig erforscht, weshalb die Studie hier einen bedeutsamen Beitrag zum Forschungsstand leistet. Die vorliegenden Ergebnisse deuten darauf hin, dass der Fokus der Unterstützung vermehrt auf die Gruppe der älteren Mitglieder gelegt werden sollte, da sie während der Pandemie mit vielen neuen Herausforderungen konfrontiert sind. Hier sind telefonische Gespräche und Beratung zur Unterstützung und Förderung der Mitglieder indiziert. Allerdings sollte langfristig eine Möglichkeit gefunden werden diese Personengruppe auch digital besser aufzustellen. Darauf macht auch die „Bundesarbeitsgemeinschaft Selbsthilfe von Menschen mit Behinderung, chronischer Erkrankung und ihren Angehörigen e. V.“ (BAG Selbsthilfe) aufmerksam und fordert Selbsthilfegruppen auf, im Rahmen der jeweiligen Bedarfe und Möglichkeiten passende Strategien zur Verbesserung der Gesundheitskompetenz ihrer Mitglieder zu entwickeln [1]. Durch Unterstützung der Menschen in vulnerablen Lebenslagen kann Selbsthilfe positiv auf die Gesundheitsversorgung und Gesundheitskompetenz der gesamten Bevölkerung wirken [4]. Dabei sollte die Unterstützung der Mitglieder unter Berücksichtigung der sozialen Unterschiede stattfinden, sodass Personen mit Schwierigkeiten gezielt gefördert werden können, um allen Mitgliedern gleiche Teilhabechancen an Gesundheit zu ermöglichen.

### Limitationen

- Informationen zur Gesundheitskompetenz der Mitglieder wurden über Fremdeinschätzung durch die Vertreter*innen der Selbsthilfe erhoben.
- Aufgrund der bestehenden Bestimmungen zur Ausbreitung des Coronavirus wurden die Interviews telefonisch durchgeführt. Nonverbale Reaktionen konnten dadurch nicht wahrgenommen werden.

## Fazit für die Praxis

- Während der Corona-Pandemie stellt gerade die gesundheitsbezogene Selbsthilfe einen wichtigen Akteur zur Stärkung der Gesundheitskompetenz ihrer chronisch erkrankten Mitglieder dar.
- Durch Schwierigkeiten im Sprachverständnis, bspw. bei Mitgliedern mit einem Migrationshintergrund oder durch die Verwendung medizinischer Fachsprache in der Kommunikation, bedarf es gezielter, verständlich aufbereiteter Angebote und Unterstützungsmöglichkeiten in verschiedenen Sprachen.
- Digitale Angebote zum Austausch und zur Weitergabe von Gesundheitsinformationen erweisen sich als hilfreich, um Selbsthilfemitglieder während der Pandemie zu unterstützen.
- Unabhängig von technischer und finanzieller Ausstattung oder dem Alter sollten Mitglieder die Möglichkeit zum Austausch aus ihrem häuslichen Umfeld bekommen.
- Ein kompetenter Umgang mit Gesundheitsinformationen, also eine gute Gesundheitskompetenz, ist insbesondere für Menschen mit Beeinträchtigung bedeutsam, da sie sich täglich mit Informationen bezüglich ihrer Therapien oder Medikamente auseinander setzten müssen

## References

[1] BAG Selbsthilfe (2019) Arbeitshilfe – Gesundheitskompetenz von chronisch kranken und behinderten Menschen stärken. https://www.bag-selbsthilfe.de/fileadmin/user_upload/_Politische_INTERESSENVERTRETUNG/Gesundheitspolitik/Praevention_Gesundheitsfoerderung/Arbeitshilfe_-_Gesundheitskompetenz_von_chronisch_kranken_und_behinderten_Menschen_staerken.docx. Zugegriffen: 11. Juli 2021

[2] Dierks ML, Kofahl C (2019) Die Rolle der gemeinschaftlichen Selbsthilfe in der Weiterentwicklung der Gesundheitskompetenz der Bevölkerung. Bundesgesundheitsbl 62:17–25. 10.1007/s00103-018-2857-1 10.1007/s00103-018-2857-1 30535945

[3] Ernstmann N, Bauer U, Berens E‑M et al (2020) DNVF Memorandum Gesundheitskompetenz (Teil 1) – Hintergrund, Relevanz, Gegenstand und Fragestellungen in der Versorgungsforschung. Gesundheitswesen 82(7):e77–e93. 10.1055/a-1191-3689 32698208 10.1055/a-1191-3689

[4] Jordan S, Hoebel J (2015) Gesundheitskompetenz von Erwachsenen in Deutschland: Ergebnisse der Studie „Gesundheit in Deutschland aktuell“ (GEDA). Bundesgesundheitsblatt Gesundheitsforschung Gesundheitsschutz 58(9):942–950 26227894 10.1007/s00103-015-2200-z

[5] Kofahl C, Haack M, Nickel S et al (Hrsg) (2019) Wirkungen der gemeinschaftlichen Selbsthilfe. Medizinsoziologie, Bd. 29. LIT, Berlin

[6] Kofahl C, Schulz-Nieswandt F, Dierks ML (Hrsg) (2016) Selbsthilfe und Selbsthilfeunterstützung in Deutschland. Medizinsoziologie, Bd. 24. LIT, Berlin

[7] Kuckartz U (2014) Mixed methods. Springer, Wiesbaden

[8] Kuckartz U (2018) Qualitative Inhaltsanalyse. Methoden, Praxis, Computerunterstützung, 4. Aufl. Grundlagentexte Methoden. Beltz Juventa, Weinheim, Basel

[9] Okan O, de Sombre S, Hurrelmann K et al (2020) Gesundheitskompetenz der Bevölkerung im Umgang mit der Coronavirus-Pandemie. Studie im Auftrag des Interdisziplinären Zentrums für Gesundheitskompetenzforschung der Universität Bielefeld und der Hertie School of Governance, durchgeführt vom Institut für Demoskopie Allensbach

[10] Rathmann K, Dadaczynski K (2020) Gesundheitskompetenz von Menschen mit Behinderung in Einrichtungen für Menschen mit Behinderung im Bereich Wohnen und Arbeiten: Ergebnisse der GeKoMB-Studie. https://fuldok.hs-fulda.de/opus4/frontdoor/index/index/docId/868. Zugegriffen: 10.07.2021

[11] Rathmann K, Nellen C (2019) Gesundheitskompetenz von Menschen mit Behinderung. Präv Gesundheitsf 14(4):375–383

[12] Schaeffer D, Berens E‑M, Gille S et al (2021) Gesundheitskompetenz der Bevölkerung in Deutschland – vor und während der Corona Pandemie. Ergebnisse des HLS-GER 2. Universität Bielefeld, Interdisziplinäres Zentrum für Gesundheitskompetenzforschung (IZGK), Bielefeld

[13] Schaeffer D, Schmidt-Kaehler S, Dierks M‑L et al (2019) Strategiepapier #2 zu den Empfehlungen des Nationalen Aktionsplans. Gesundheitskompetenz in die Versorgung von Menschen mit chronischer Erkrankung integrieren. Nationaler Aktionsplan Gesundheitskompetenz, Berlin

[14] Schaeffer D, Vogt D, Berens E‑M et al (2016) Gesundheitskompetenz der Bevölkerung in Deutschland. Ergebnisbericht. Universität Bielefeld, Bielefeld

